# Age- and Gender-Related Differences in the Geometric Properties and Biomechanical Significance of Intracortical Porosity in the Distal Radius and Tibia

**DOI:** 10.1359/jbmr.091104

**Published:** 2009-11-02

**Authors:** Andrew J Burghardt, Galateia J Kazakia, Sweta Ramachandran, Thomas M Link, Sharmila Majumdar

**Affiliations:** 1 Musculoskeletal Quantitative Imaging Research Group, Department of Radiology and Biomedical Imaging, University of California–San Francisco San Francisco, CA, USA; 2 Department of Bioengineering, University of California–Berkeley Berkeley, CA, USA

**Keywords:** HR-PQCT, cortical bone, cortical porosity, osteoporosis, Micro-computed tomography

## Abstract

Cortical bone contributes the majority of overall bone mass and bears the bulk of axial loads in the peripheral skeleton. Bone metabolic disorders often are manifested by cortical microstructural changes via osteonal remodeling and endocortical trabecularization. The goal of this study was to characterize intracortical porosity in a cross-sectional patient cohort using novel quantitative computational methods applied to high-resolution peripheral quantitative computed tomography (HR-pQCT) images of the distal radius and tibia. The distal radius and tibia of 151 subjects (57 male, 94 female; 47 ± 16 years of age, range 20 to 78 years) were imaged using HR-pQCT. Intracortical porosity (Ct.Po) was calculated as the pore volume normalized by the sum of the pore and cortical bone volume. Micro–finite element analysis (µFE) was used to simulate 1% uniaxial compression for two scenarios per data set: (1) the original structure and (2) the structure with intracortical porosity artificially occluded. Differential biomechanical indices for stiffness (Δ*K*), modulus (Δ*E*), failure load (Δ*F*), and cortical load fraction (ΔCt.LF) were calculated as the difference between original and occluded values. Regression analysis revealed that cortical porosity, as depicted by HR-pQCT, exhibited moderate but significant age-related dependence for both male and female cohorts (radius ρ = 0.7; tibia ρ = 0.5; *p* < .001). In contrast, standard cortical metrics (Ct.Th, Ct.Ar, and Ct.vBMD) were more weakly correlated or not significantly correlated with age in this population. Furthermore, differential µFE analysis revealed that the biomechanical deficit (Δ*K*) associated with cortical porosity was significantly higher for postmenopausal women than for premenopausal women (*p* < .001). Finally, porosity-related measures provided the only significant decade-wise discrimination in the radius for females in their fifties versus females in their sixties (*p* < .01). Several important conclusions can be drawn from these results. Age-related differences in cortical porosity, as detected by HR-pQCT, are more pronounced than differences in standard cortical metrics. The biomechanical significance of these structural differences increases with age for men and women and provides discriminatory information for menopause-related bone quality effects. © 2010 American Society for Bone and Mineral Research.

## Introduction

Osteoporosis is characterized by loss of bone mass and compromised bone structure resulting in reduced bone strength and an increased risk of fracture. The standard clinical method for assessing bone mass, areal bone mineral density (aBMD) determined by dual-energy X-ray absorptiometry (DXA), does not entirely explain fracture risk. This X-ray projection technique does not account for 3D geometry and obfuscates independent effects in cortical and cancellous bone compartments.(1–4) Accordingly, the development of quantitative endpoints based on 3D imaging techniques is an important goal toward an improved mechanistic understanding of bone mechanics, fracture risk, disease progression, and therapeutic efficacy.

Micro-computed tomography (µCT) has become an important tool for investigating a wide range of aspects related to the biology of bone and other calcified tissues.(5–7) Until recently, true 3D in vivo assessment of bone microstructure has been limited to small-animal microtomographic systems.(8,9) High-resolution peripheral quantitative computed tomography (HR-pQCT) is a promising noninvasive method for in vivo 3D characterization of bone in humans.(10,11) The most recent generation of this device is capable of an isotropic nominal resolution of 82 µm. This permits quantification of the geometric, microstructural, densitometric, and mechanical properties of human cortical and trabecular bone in the appendicular skeleton (distal radius and tibia).(12–14)

Thus far, clinical HR-pQCT studies have focused primarily on trabecular microstructure.(15–17) However, there is considerable evidence that the cortical bone bears the bulk of axial loads in the distal radius and tibia(18) and that the distribution of load is an important factor in bone strength and fracture prediction.(19,20) Consistent with these observations, both gross geometric and microstructural properties of cortical bone are known to contribute to overall bone strength.(21) These properties are modified through distinct physiologic processes (endosteal/periosteal remodeling versus osteonal remodeling) and therefore represent unique targets for pharmacologic intervention. Recently, evidence of significant variability in the morphologic manifestation of bone loss in the peripheral skeleton—including macroscopic intracortical porosity—has been reported for early postmenopausal women.(16) A noninvasive technique to quantitatively evaluate cortical microstructural features would add an important endpoint for clinical osteoporosis research. In combination with measures of cross-sectional geometry and trabecular microstructure, treatment selection could be tailored to specific bone deficit patterns (eg, cortical porosity, cortical thinning, trabecular thinning, etc.).

While the resolution of HR-pQCT is not sufficient to accurately depict cortical porosity at the level of single normal haversian and Volkmann canals, we hypothesize that the resolvable level of intracortical porosity changes with age in coincidence with an overall decrease in bone mass. The goal of this study was to assess age- and gender-related differences in intracortical porosity of the distal radius and tibia. Furthermore, the biomechanical significance of intracortical porosity was evaluated using micro–finite element (µFE) analyses in image data sets with natural cortical porosity and in simulated data sets with the natural cortical porosity digitally occluded.

## Materials and Methods

### Subjects

HR-pQCT image data from the baseline examinations of an ongoing cross-sectional patient study were evaluated for this study. The subjects consisted of 151 male and female volunteers (female *n* = 97, age = 48.5 ± 15.7 years, male *n* = 54, age = 45.1 ± 16.5 years) spanning a wide range of ages (20 to 78 years) and anthropometrics [body mass index (BMI) range 17 to 39; female (*n* = 48 subset): height = 161 ± 7 cm, weight = 65 ± 16 kg; male (*n* = 20 subset): height = 175 ± 8 cm, weight = 79 ± 16 kg). Of the subjects, 46% were white, 45% were Asian, 6% were Hispanic, and 3% were African American—approximately reflecting the diverse ethnic composition of the San Francisco Bay Area. The mean age of subjects in each ethnic group was not statistically different. For this study, DXA screening was not performed prior to enrollment; therefore, no bone mineral density (BMD) inclusion/exclusion criteria were used. History of or evidence for metabolic bone disease other than postmenopausal bone loss was an exclusion criterion. The study protocol was approved by the UCSF Committee on Human Research, and all subjects gave written informed consent prior to participation.

### HR-pQCT imaging

All subjects were imaged in a clinical HR-pQCT system (XtremeCT, Scanco Medical AG, Brüttisellen, Switzerland) using the manufacturer's standard in vivo protocol described in previous patient studies.(15–17) The subject's forearm/ankle was immobilized in a carbon fiber cast that was fixed within the gantry of the scanner. A single dorsal-palmar projection image of the distal radius/tibia was acquired to define the tomographic scan region. This region spanned 9.02 mm in length (110 slices) and was fixed starting at 9.5 and 22.5 mm (for the radius and tibia, respectively) proximal from the middle jointline and extending proximally. For tomography, 750 projections were acquired over 180 degrees with a 100-ms integration time at each angular position. The 12.6-cm field of view (FOV) was reconstructed across a 1536 × 1536 matrix using a modified Feldkamp algorithm, yielding 82-µm voxels.(21) Total scan time was 2.8 minutes with an equivalent dose of approximately 4.2 µSv for each site. Of the 151 subjects examined, 4 had only a radius scan, 5 had only a tibia scan, and 5 forearm acquisitions were excluded owing to excessive motion artifacts. As a result, a total of 142 radii (87 female, 55 male) and 146 tibiae (93 female, 53 male) ultimately were evaluated (Table 1).

**Table 1:** Summary of Subject Numbers by Decade

Decade							
Sex	Site	20	30	40	50	60	70
Female	Radius	15	14	12	22	17	7
	Tibia	17	16	14	21	18	7
Male	Radius	12	13	6	13	8	3
	Tibia	13	13	5	11	8	3

### Image analysis

**Fig. 1:**
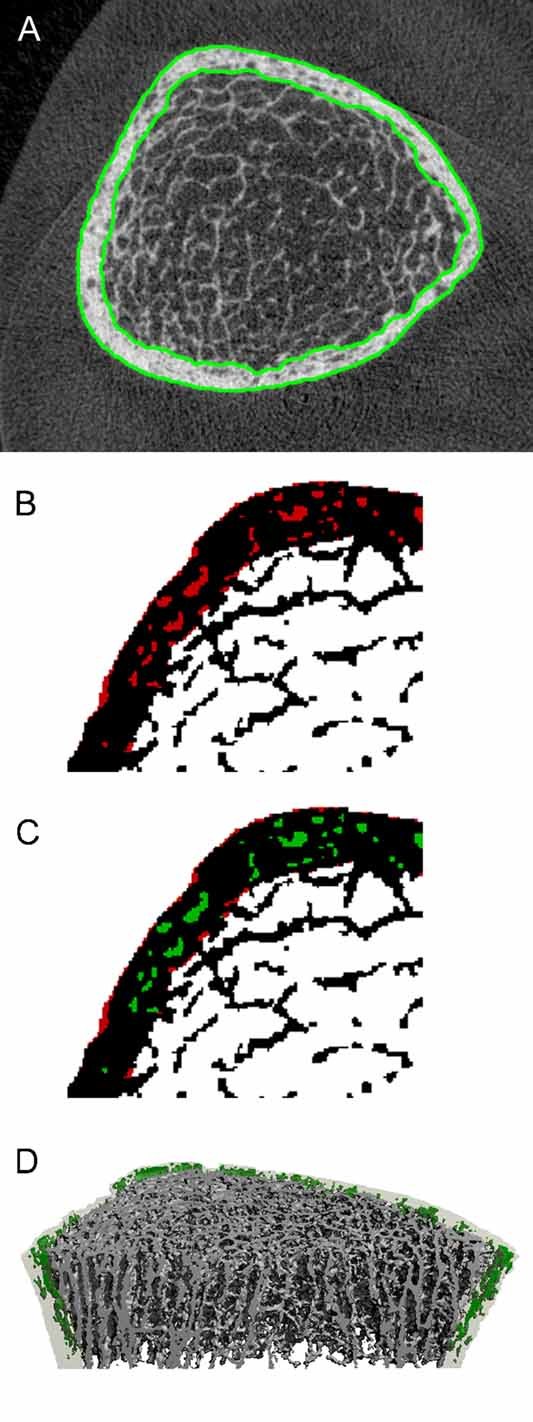
Representative 2D image showing cortical compartment contour (*A*) and segmented cortical void (red) (*B*). (*C*) Image demonstrating segmentation of intracortical porosity (green) from endosteal/periosteal void (*red*). (*D*) 3D surface rendering of intracortical components within the native bone structure.

All image analysis was performed in a custom-built Image Processing Language (IPL Version 5.06a-ucsf, Scanco Medical AG) that includes in-house-developed functionality. For reference, simulated areal bone mineral density (aBMD_sim_) was calculated from the HR-pQCT images for the radius. This technique was established previously to approximate DXA aBMD measures with accuracy comparable with intermanufacturer differences (eg, Hologic versus Prodigy).(22) Additionally, the images were processed using the default clinical evaluation protocol provided by the manufacturer to derive standard cortical geometric and density measures.(10) This included cortical area (Ct.Ar), cortical thickness (Ct.Th), cortical volumetric bone mineral density (Ct.vBMD), and total volumetric bone mineral density (vBMD). Subsequent image processing and analysis were based on the binary image generated by the standard protocol.(10) This process segments the mineralized and background phases of fine structures using a simple fixed threshold following the application of a Laplace-Hamming edge-enhancement filter. Previously, this method was optimized for trabecular bone segmentation of images acquired with a lower-resolution pQCT device(23) and subsequently has been shown to provide good accuracy for HR-pQCT.(12,13) Acceptable application of this technique for the segmentation of fine cortical structure was qualitatively verified by visual inspection in a series of data sets spanning a range of geometries. The periosteal and endosteal boundaries were defined using an automated contouring method similar to the technique described previously by Buie and colleagues.(24) The region between the two contours was considered the cortical compartment volume of interest (VOI) (Fig. 1*A*). Owing to the imprecise nature of the cortical compartment segmentation, this VOI typically includes void voxels from the background on the periosteal and endosteal margins as well as intracortical porosity (Fig. 1*B*). To exclusively segment the intracortical porosity volume, a novel algorithm based on 2D component labeling and a 3D region-growing process was applied: True intracortical porosity was estimated initially to be all void voxels unconnected to the background in each 2D axial slice. Next, a region-growing process additionally included void voxels connected along the *z* axis (SI direction) to the initial pore voxels (Fig. 1*C, D*). Based on this segmentation, cortical porosity (Ct.Po) was defined as a normalized volumetric index according to Eq. (1):


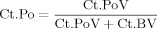
(1)

where Ct.PoV is the segmented pore volume, and Ct.BV is the mineralized cortical bone volume. In this way, variability related to cortical compartment segmentation and endosteal and periosteal surface irregularities did not bias the measure.

### µFE analysis

Linear µFE analysis was applied to calculate apparent biomechanical properties under uniaxial compression. Two models were created for each subject scan: (1) a model based on the standard segmentation process (original) and (2) a model where the segmented intracortical porosity was digitally occluded, that is, converted from void elements to bone elements (occluded). Homogeneous mechanical properties were assumed for all bone elements. The binary image data set was converted to a mesh of isotropic brick elements using a voxel conversion technique,(25) and each element was assigned an elastic modulus of 10 GPa(26) and a Poisson ratio of 0.3.(27) Cortical and trabecular bone elements were labeled as different materials with identical material properties to facilitate calculation of compartmental load distribution. On average, the models were composed of 1,970,000 and 4,610,000 elements for the original radius and tibia data sets, respectively. A uniaxial compression test in the axial direction (superoinferior) was performed with an applied strain of 1%. An iterative solver (Scanco FE Software Version 1.12, Scanco Medical) was used to compute reaction forces at the superior and inferior ends of the sections for the prescribed boundary conditions. The model computations were performed at the UCSF/QB3 Shared Computing Facility, a mixed-architecture Linux HPC grid consisting of 1900 processor nodes. The models required an average of 2.4 and 6.3 CPU hours for the original radius and tibia data sets, respectively, and required 2820 CPU hours in total for the 576 models. No models were excluded owing to convergence limitations.

**Fig. 2:**
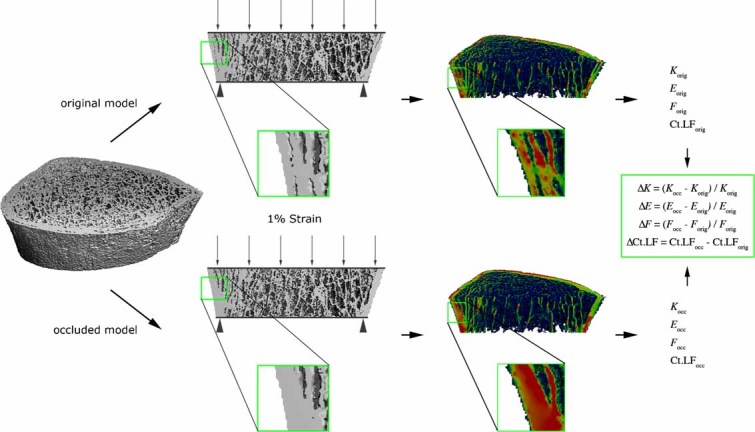
Schematic for the differential µFE modeling process. Two models are computed: the first from the original image data (*top*) and the second from a model with the intracortical pore space digitally occluded—that is, given bone-equivalent material properties (*bottom*). The relative difference in the computed mechanical properties is understood to be the mechanical deficit attributable to the cortical porosity, as depicted by HR-pQCT. The pseudo-color renderings show the distribution of strain energy density (SED) for each model.

For each model, stiffness (*K*), apparent modulus (*E*), and the load fraction for the cortical compartment (Ct.LF) were calculated. Furthermore, failure load (*F*) was estimated using methods previously described by Pistoia and colleagues.(28) Differential biomechanical indices were calculated from each original and occluded model pair (Fig. 2). Specifically, the deficit in stiffness (Δ*K*), apparent modulus (Δ*E*), and failure load (Δ*F*) owing to the resolvable intracortical porosity were calculated as the difference in the respective parameter between the occluded and original models and normalized by the original. These indices were reported as a percent. The fractional load (ΔCt.LF) shifted from the cortex to the trabecular bone was calculated simply as the difference between occluded and original Ct.LF.

### Statistical analysis

Mean and standard deviation were calculated for all indices and were determined for each site and gender. Since the Shapiro-Wilk *W* test revealed that multiple parameters were not normally distributed, all gender and site differences across the pooled data set and within each decade were evaluated for statistically significant differences using the Wilcoxon rank-sum test with α = 0.05. Female subjects were divided into pre- and postmenopausal cohorts, and the percent difference for each parameter was calculated as the difference between the means for pre- and postmenopausal cohorts divided by the premenopausal mean. Significant differences in cortical bone quality measures between pre- and postmenopausal cohorts were evaluated using the Wilcoxon rank-sum test with α = 0.05. Age-related differences were evaluated on a per-decade basis using Kruskal-Wallis one-way ANOVA followed by a Wilcoxon rank-sum test for pairwise comparisons across consecutive decades. To account for multiple comparisons in the decade-wise analysis, a Bonferroni correction was applied. Accordingly, the level of statistical significance was set to *p* < .01. Finally, Spearman correlations were performed to evaluate age-related effects for a given parameter for each gender and site.

## Results

**Fig. 3:**
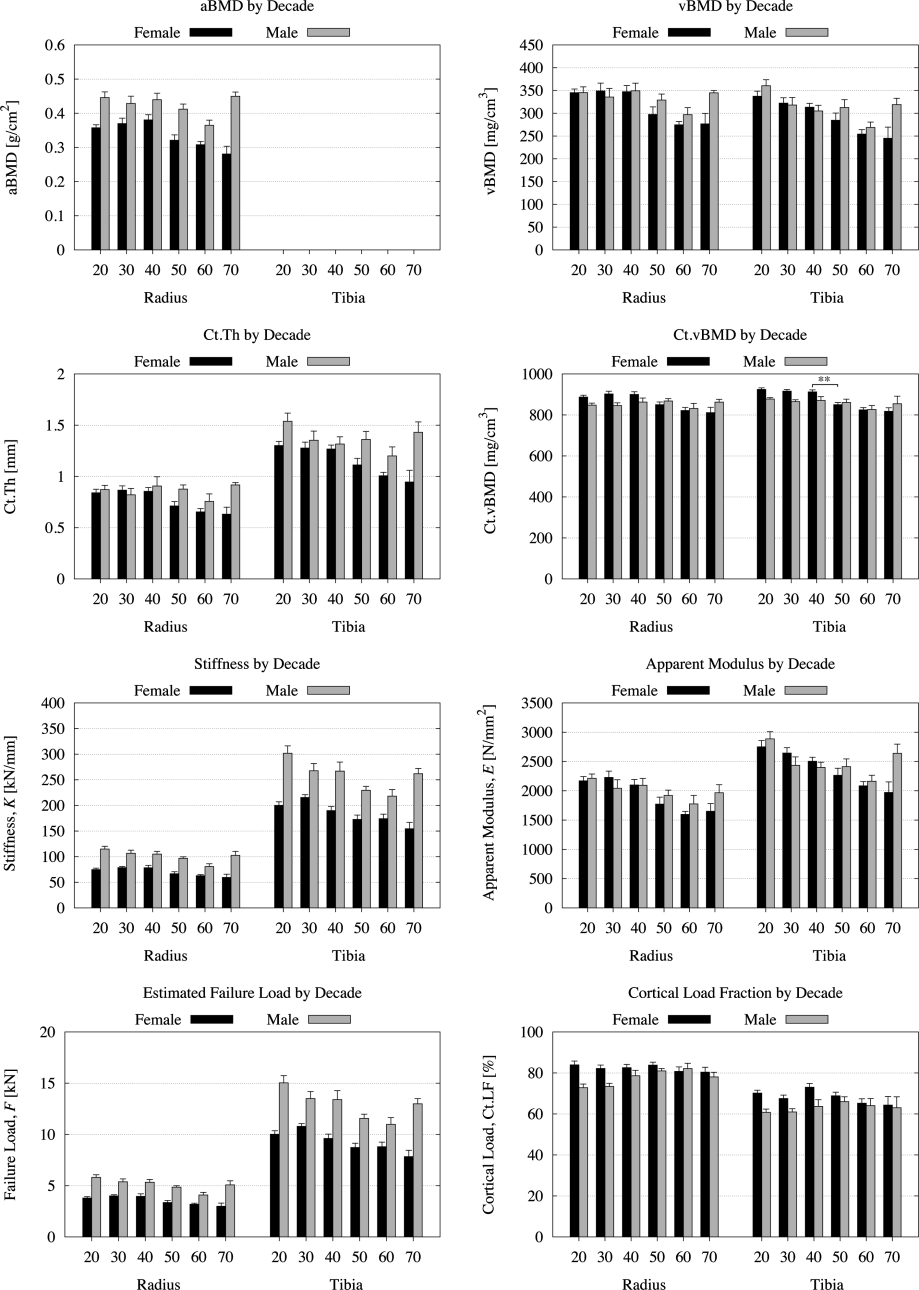
Decade-wise trends for selected standard density, geometric, and biomechanical parameters. Bars represent mean with standard error indicated for female (*black*) and male (*gray*) subjects. Statistical significance between consecutive decades (Wilcoxon ranked sum with Bonferroni correction) is denoted by ^*^p < .01 and ^**^p < .001).

**Fig. 4:**
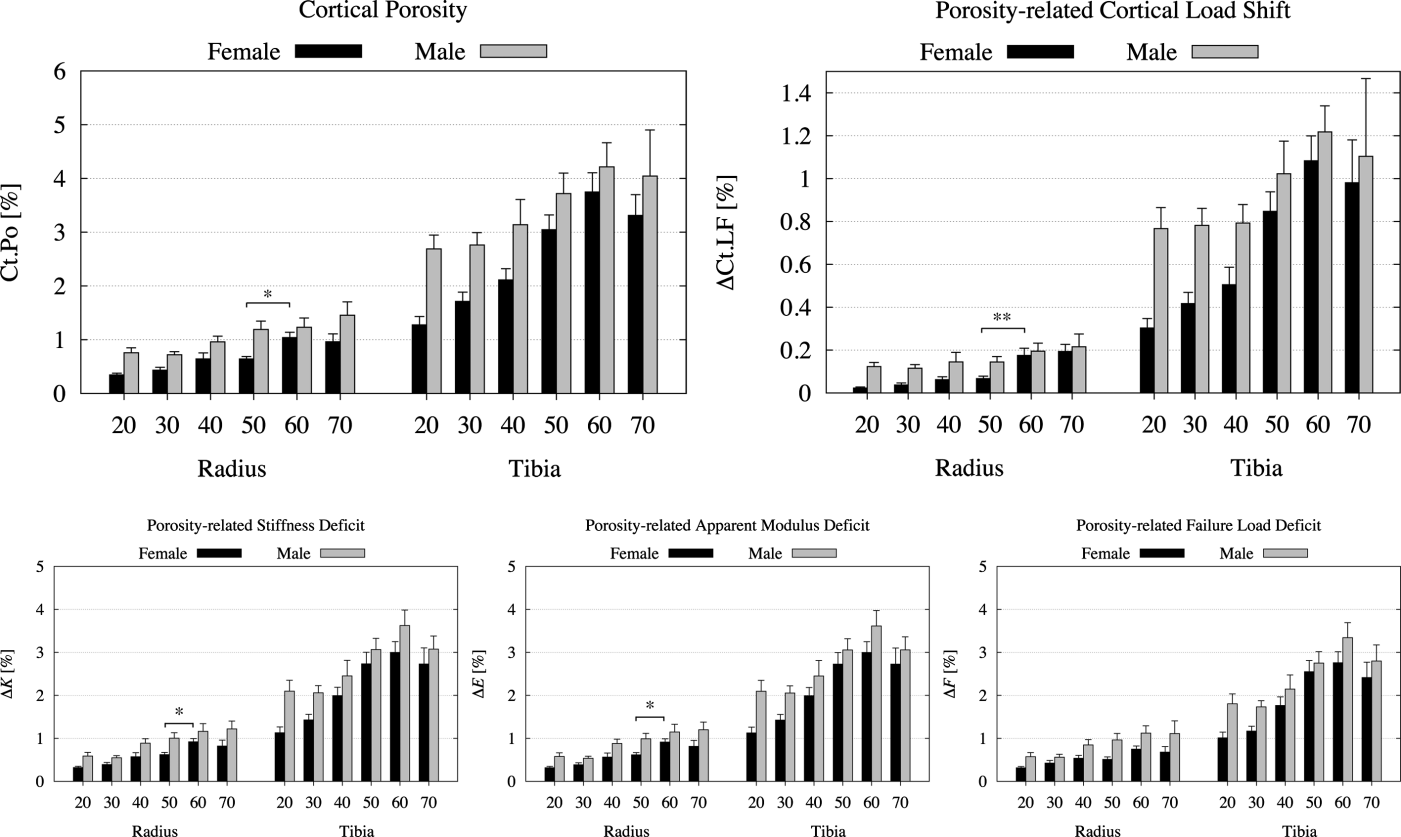
Decade-wise trends for cortical porosity and porosity-related biomechanical parameters. Bars represent mean with standard error indicated for female (*black*) and male (*gray*) subjects. Statistical significance between consecutive decades (Wilcoxon ranked sum with Bonferroni correction) is denoted by ^*^p < .01 and ^**^p < 0.001).

The mean and standard deviations for all cortical measures are presented in Table 2 by gender and site. The Spearman correlation coefficients for the regression analysis are similarly presented in Table 3. Decade-wise comparisons for the standard cortical densitometric, geometric, and biomechanical parameters are presented in bar-graph form in Fig. 3, whereas Ct.Po and the differential µFE results are presented in decade-wise fashion in Fig. 4. Finally, pre- and postmenopause comparisons for all parameters are presented in Fig. 5.

**Table 2:** Summary of Measures by Gender and Site: Mean (SD)

	Female		Male	
		
Measure	Radius (*n* = 87)	Tibia (*n* = 93)	Radius (*n* = 55)	Tibia (*n* = 53)
*Standard measures*				
aBMD_sim_ (g/cm^2^)	0.34 (0.06)^a^	—	0.42 (0.06)^a^	—
vBMD (g/cm^3^)	0.31 (0.06)	0.30 (0.06)^d^	0.33 (0.05)	0.32 (0.06)^d^
Ct.Ar (mm^2^)	50.2 (11.1)^a^	113.7 (21.4)^a^	67.7 (13.8)^a^	156.8 (33.2)^a^
Ct.Th (mm)	0.76 (0.18)^c^	1.17 (0.25)^a^	0.85 (0.18)^c^	1.38 (0.28)^a^
Ct.vBMD (g/cm^3^)	0.86 (0.06)	0.88 (0.06)^d^	0.85 (0.05)	0.86 (0.04)^d^
*Biomechanical measures*				
Stiffness, *K* (kN/mm)	70.2 (14.9)^a^	186.6 (36.1)^a^	101.9 (19.4)^a^	260.2 (50.9)^a^
Modulus, *E* (N/mm^2^)	1914 (446)	2398 (486)	2013 (388)	2506 (468)
Estimated failure load, *F* (N)	3552 (763)^a^	9398 (1790)^a^	5138 (941)^a^	13050 (2491)^a^
Ct.LF (%)	82.4 (7.0)^a^	68.4 (8.0)^a^	77.2 (6.7)^a^	62.8 (7.2)^a^
*Porosity measures*				
Ct.Po (%)	0.7 (0.4)^a^	2.5 (1.4)^b^	1.0 (0.5)^a^	3.3 (1.2)^b^
Δ*K* (%)	0.6 (0.3)^c^	2.2 (1.1)^c^	0.8 (0.4)^c^	2.6 (1.0)^c^
Δ*E* (%)	0.6 (0.3)^c^	2.2 (1.1)^c^	0.8 (0.4)^c^	2.6 (1.0)^c^
Δ*F* (%)	0.5 (0.3)^b^	1.9 (1.1)^c^	0.8 (0.4)^b^	2.3 (0.9)^c^
ΔCt.LF (%)	0.1 (0.1)^a^	0.7 (0.5)^b^	0.1 (0.1)^a^	0.9 (0.4)^b^

a Denotes *p* < .0001 female versus male.

b Denotes *p* < .001 female versus male.

c Denotes *p* < .01 female versus male.

d Denotes *p* < .05 female versus male.

**Table 3:** Spearman's Correlation Coefficients (ρ) for Each Measure Against Age*

	Female		Male	
		
Measure	Radius (*n* = 87)	Tibia (*n* = 93)	Radius (*n* = 55)	Tibia (*n* = 53)
*Standard measures*				
aBMD_sim_ (g/cm^2^)	−.50	—	−.30^c^	—
vBMD (g/cm^3^)	−.55	−.57	NS	−.48^a^
Ct.Ar (mm^2^)	−.43	−.50	NS	−.31^c^
Ct.Th (mm)	−.50	−.55	NS	NS
Ct.vBMD (g/cm^3^)	−.49	−.71	NS	−.29^c^
*Biomechanical measures*				
Stiffness, *K* (kN/mm)	−.46	−.49	−.48^a^	−.52
Modulus, *E* (N/m^2^])	−.62	−.58	−.36^b^	−.41^b^
Estimated failure load, *F* (N)	−.47	−.48	−.50	−.52
Ct.LF (%)	NS	NS	.48^a^	NS
*Porosity measures*				
Ct.Po (%)	.70	.73	.52	.51
Δ*K* (%)	.67	.69	.57	.57
Δ*E* (%)	.67	.70	.57	.57
Δ*F* (%)	.51	.70	.48^a^	.59
ΔCt.LF (%)	.69	.68	.30^c^	.40^b^

* Statistical significance *p* < .0001 unless not significant (NS) or denoted otherwise.

a Denotes *p* < .001.

b Denotes *p* < .01.

c Denotes *p* < .05.

### Cortical porosity in the radius and tibia

Intracortical porosity in the tibia (range 0.3% to 7.1%) was approximately threefold higher than in the radius (range 0.2% to 2.4%, *p* < .0001). Similarly, the apparent mechanical deficits attributed to intracortical porosity were significantly higher in the tibia (range 0.3% to 6.0% for Δ*K*) compared with the radius (range 0.1% to 2.0% for Δ*K*, *p* < .0001). In the radius, these deficits were associated with a transfer of load from the cortical compartment to the trabecular compartment (ΔCt.LF) ranging from 0.0% to 0.5%, whereas the tibia ranged from 0.0% to 2.2% (*p* < .0001).

### Gender differences in cortical bone quality

In general, gender-based differences were comparable for the radius and tibia (Figs. 3 and 4). Total and cortical vBMD values were approximately equivalent between male and female cohorts, with Ct.vBMD only significantly different between young males and females in the radius (20s: *p* < .01; 30s: *p* < .01) and tibia (20s and 30s: *p* < .001). However, aBMD of the radius was significantly lower in women (*p* < .0001). When pooled across all decades, male subjects were found to have a larger cortical area (*p* < .0001) and thicker cortices than female subjects (radius: *p* < .01; tibia: *p* < .0001). The gender-wise difference in Ct.Th tended to be significant in elderly subjects (>50 years, *p* < .05 except 60s radius and 70s tibia) and not for the younger cohorts. Size-dependent biomechanical indices (ie, stiffness, estimated failure load) were significantly higher in male subjects overall (tibia and radius: *p* < .0001), as well as within each decade cohort. In contrast, apparent modulus, which is size-independent, was not different between genders. Cortical load fraction (Ct.LF) was significantly higher for women than for men pooled across all ages (*p* < .0001), although statistically significant differences were limited to younger subjects in the decade-wise cohort analysis (radius: 20s, 30s; tibia: 20s, 30s, 40s; *p* < .05). All porosity-related measures showed significant differences between genders (*p* < .01). Greater sexual dimorphism generally was found in the younger cohorts, with males having significantly greater Ct.Po in their 20s and 30s than females (*p* < .01), whereas the differences were more muted from 50 years onward. This was marked by a dramatic increase in Ct.Po for women—threefold from the 20s to the 60s (radius and tibia: *p* < .0001)—whereas males increased 60% over the same period (radius: *p* < .01; tibia: *p* < .05).

### Age-related differences in cortical bone quality

Linear regression analysis revealed moderate, statistically significant correlations with age for all standard densitometric and geometric parameters in the distal radius and tibia of female subjects (all *p* < .0001). With the exception of aBMD_sim_ (*p* < .05), none of the standard density and geometric measures showed statistically significant age-related correlations in the radius of the male cohort. Weak but statistically significant correlations with age were found for total vBMD, Ct.Ar, and Ct.vBMD (ρ = −0.29 to −0.48) in the tibia. The largest consecutive decade-wise differences for standard density, geometric, and biomechanical indices typically were found between the 40s and the 50s cohorts in women, consistent with the onset of menopause. The apparent biomechanical properties of the tibia and radius for both genders exhibited moderate and statistically significant correlations with age (ρ = −0.36 to −0.62). Only the radius of male subjects exhibited statistically significant correlations with age for cortical load fraction (ρ = −0.48). Cortical porosity and associated differential biomechanical deficit measures (Δ*K*, Δ*E*, and Δ*F*) exhibited moderate to high age-related correlations (ρ = +0.48 to +0.73), whereas ΔCt.LF was moderately well correlated with age in females (ρ = +0.68) and weakly in males (radius: ρ = +0.30; tibia: ρ = +0.40). In female subjects, the largest single-decade difference in cortical porosity was found to be between the 50s and the 60s in the radius (*p* < .01) and the 40s and the 50s in the tibia (not significant). In general, male subjects did not exhibit pronounced shifts in cortical bone measures across specific decades.

### Postmenopausal differences in cortical bone quality

The female subjects were further divided into young and elderly cohorts based on menopausal status. All postmenopausal women were 50 years of age or older, and (with the exception of one 50-year-old) all premenopausal subjects were younger than 50 years of age. As summarized in Table 4, postmenopausal subjects had significantly lower bone quality indices (*p* < .0001). Only the cortical load fraction (Ct.LF) was not statistically different between pre- and postmenopause cohorts. Ct.Po and associated differential µFE indices all were found to have significant differences between pre- and postmenopausal subjects (all > 50%, *p* < .0001).

**Table 4:** Pre- Versus Postmenopause Differences*

	Radius			Tibia		
		
Measure	Pre (*n* = 42)	Post (*n* = 44)	% Difference	Pre (*n* = 47)	Post (*n* = 46)	% Difference
*Standard measures*						
aBMD_sim_ (g/cm^2^)	0.37 (0.05)	0.31 (0.06)	15.9%	—	—	—
vBMD (g/cm^3^)	0.35 (0.05)	0.28 (0.06)	17.9%	0.32 (0.04)	0.27 (0.06)	16.4%
Ct.Ar (mm^2^)	55.4 (9.1)	45.6 (10.8)	17.8%	123.6 (17.0)	103.6 (20.8)	16.2%
Ct.Th (mm)	0.85 (0.14)	0.67 (0.17)	21.2%	1.27 (0.19)	1.06 (0.26)	17.2%
Ct.vBMD (g/cm^3^)	0.89 (0.04)	0.83 (0.06)	6.9%	0.92 (0.03)	0.84 (0.05)	8.7%
*Biomechanical measures*						
Stiffness, *K* (kN/mm)	77.2 (12.5)	63.7 (14.2)	17.5%	202.1 (28.8)	170.7 (36.1)	15.5%
Modulus, *E* (N/mm^2^)	2166 (368)	1679 (386)	22.5%	2614 (388)	2176 (481)	16.7%
Estimated failure load, *F* (N)	3919 (644)	3208 (717)	18.1%	10154 (1421)	8626 (1811)	15.0%
Ct.LF (%)	83.0 (6.2)	82.0 (7.7)	1.2%	70.1 (6.7)	66.8 (9.0)	NS
*Porosity measures*						
Ct.Po (%)	0.46 (0.27)	0.86 (0.37)	−87.6%	1.68 (0.76)	3.35 (1.35)	−99.2%
Δ*K* (%)	0.42 (0.23)	0.77 (0.31)	−85.3%	1.50 (0.68)	2.83 (1.15)	−88.7%
Δ*E* (%)	0.41 (0.23)	0.77 (0.31)	−85.6%	1.50 (0.68)	2.82 (1.15)	−88.8%
Δ*F* (%)	0.41 (0.21)	0.63 (0.30)	−53.3%	1.30 (0.66)	2.60 (1.16)	−100.9%
ΔCt.LF (%)	0.04 (0.04)	0.13 (0.11)	−232.7%	0.41 (0.24)	0.96 (0.46)	−136.2%

* All statistical significance *p* < .0001 unless not significant (NS).

## Discussion

In this study we have determined age- and gender-related differences in intracortical porosity of the distal radius and tibia using in vivo HR-pQCT measurements in human subjects. Furthermore, the biomechanical significance of these features, and its evolution with age, has been investigated using differential µFE analyses. In general, cortical porosity was found to increase with age in both men and women in coincidence with a decline in bone mass, as measured by aBMD and vBMD. Furthermore, the biomechanical deficit attributable to cortical porosity was observed to increase significantly with age. It was determined that Ct.Po and associated differential FE indices show stronger correlations with age in both female and male subjects compared with standard cortical density, geometric, or biomechanical measures and that they differentiate pre- and postmenopausal women.

The primary strengths of this study are threefold. First, we have evaluated age- and gender-related cortical bone effects in a wide range of subjects. Unlike other recently published cross-sectional HR-pQCT studies,(15,17,29) our subject cohort spanned a much broader range of ethnicities, including white, Hispanic, Asian, and African-American subjects. Nevertheless, the novel cortical microstructure and biomechanical properties evaluated in this study exhibited significant age- and gender-related effects. Furthermore, the cortical porosity analysis applied in this study is unique in that it specifically quantifies intracortical pore volume and excludes periosteal and endosteal void in the cortical VOI (Fig. 1). This intracortical void presumably corresponds to osteonal remodeling sites as opposed to endocortical remodeling and real or artifactual irregularities in the endosteal or periosteal boundaries included by the cortical compartment segmentation process. Finally, we have proposed differential biomechanical indices, based on FE analysis that characterize the biomechanical significance of intracortical porosity as depicted by HR-pQCT. Specifically, the relative deficit in whole-bone mechanical properties was estimated by creating two unique models for each patient scan: a model based on the natural structure and a model with the natural intracortical porosity digitally occluded. From these models, relative differences in stiffness, modulus, and the estimated failure load attributable to the intracortical porosity are calculated. Additionally, the difference in the cortical load fraction is calculated. This measure can be interpreted as the fraction of the total axial load shifted from the cortex to the trabecular compartment owing to the intracortical pore structure.

The functional significance of cortical geometry and microstructure is readily apparent from our results. Consistent with several recent studies, the cortical compartment was found to support the large majority of axial loads in the peripheral skeleton (radius 80%, tibia 66%).(18,19,29) The differential µFE results demonstrate that intracortical porosity is associated with stiffness deficits exceeding 7% in some elderly subjects from this normal population. While the magnitudes of these mechanical deficits are relatively modest, it is important to recognize that they are associated with very minor differences in overall bone mass—Ct.Po of 2.7% in the tibia is approximately equivalent to 0.5% of total bone volume. Furthermore, they represent values from a population screened against diagnosed bone metabolic disorders. Significantly higher levels of intracortical porosity in the peripheral skeleton have been qualitatively described elsewhere(16,24) and would be expected to manifest greater deficits. Additionally, while porosity-related strength deficits were, on average, 0.7% in the radius and 2.3% in the tibia, this translated to just a 0.1% and 0.8% shift in the cortical/trabecular load distribution for the radius and tibia, respectively. This finding suggests that deleterious effects in the cortex minimally alter trabecular load burden and reinforces the significance of cortical bone changes for whole-bone strength. Consistent with this interpretation, Kirmani and colleagues observed that a transient increase in cortical porosity in the distal radius of adolescents is concurrent with the age for peak incidence of forearm fracture, whereas trabecular bone parameters were either not changing or actually increasing over the same period.(30)

An important finding of this study is that Ct.Po and the differential biomechanical indices were the only parameters to successfully differentiate female subjects in consecutive decades. Interestingly, these differences were observed in the radius between females in their 50s and 60s (*p* < .01). This coincided with significant differences in porosity-related mechanical deficit indices in the radius (*p* < .01, except Δ*F*). In contrast, the largest difference in tibial Ct.Po was observed between the previous decades (40s and 50s) and was consistent with the largest magnitude in differences for other cortical parameters in the tibia, which were not statistically significant. The latter finding is consistent with perimenopausal changes in bone mass. A possible explanation for the former result is that in the radius, perimenopausal changes in intracortical porosity do in fact occur, but the resolution limitations of HR-pQCT are insufficient to detect these structural features until later in their progression. This hypothesis is supported by the observation that radial Ct.vBMD, itself a reflection of all cortical porosity (not just macroscopic pores), as well as aBMD and vBMD, had the largest decade-wise difference between the 40s and 50s female cohorts.

There are several limitations to this study that must be addressed. First, it is apparent that the resolution constraints of HR-pQCT significantly limit the size of pores captured by this technique. Accordingly, Ct.Po, as measured here, may not reflect effects in the true porosity at the ultrastructural level. The assumption made in this analysis is that the resolvable porosity represents large, single osteonal canals or even merged adjacent osteonal canals. Accordingly, increases in osteonal diameter through excessive remodeling would be manifested by an increase in Ct.Po. Second, the differential µFE indices presented in this study were derived by fixing all geometric and material properties with the exception of the resolvable intracortical porosity. While this has the advantage of controlling all other factors contributing to bone strength in order to provide specific insight into the biomechanical role of intracortical porosity, it does not account for the integral nature of bone adaptation. Changes in intracortical porosity would be expected to be concurrent with other adaptations in cortical and trabecular structure and material properties. Accordingly, the mechanical deficits attributable to intracortical porosity, as estimated here, represent a simplification of a complex process. It remains to be seen whether limited-resolution in vivo estimates of cortical porosity and their biomechanical significance via differential µFEA provide fracture discrimination/prediction capabilities or are measurably affected by therapeutic intervention.

Segmentation of intracortical porosity initially requires an accurate segmentation of the cortical compartment. The manufacturer's method involves a semimanual contouring process followed by a simple thresholding scheme. Kirmani and colleagues used this approach to make simple estimations of cortical porosity in HR-pQCT images of adolescents.(30) However, because this segmentation process has been demonstrated to be relatively crude for thin or highly porous cortices,(16,24) we deemed it not suitable for postmenopausal osteoporosis applications. Instead, we adapted an automated method proposed by Buie and colleagues.(24) While automatic detection of the periosteal boundary is a relatively trivial process given the sharp, well-defined intensity gradient at this boundary, detection of the endosteal boundary is complicated by the presence of trabecular bone. A single method to identify the endosteal boundary in young, healthy individuals with dense trabecular bone adjacent to the cortex and elderly, osteopenic/osteoporotic individuals with thin cortices and endocortical trabecularization presents a significant challenge. As a result, some parameter adjustment was required for several patients with particularly poor cortical bone structure to achieve an acceptable segmentation. While the automated method used here was observed to be qualitatively superior to the default manufacturer's method, further improvements to eliminate segmentation failures are needed.

Several statistical limitations to the study should be noted. The sample sizes for some age groups in the decade-wise analysis were limited. As a result, age- and gender-related differences for these groups may be statistically underpowered. In particular, the eldest male and female groups (70 to 79 years) and the 40- to 49-year male group were underrepresented in the study. While an important strength of this study is the inclusion of subjects from multiple ethnic backgrounds, it is possible that interethnic differences are confounding. While the mean ages for each ethnicity were not statistically different, they were not necessarily balanced across all age subcohorts. Pooled over all ages, only cortical load fraction was statistically different between Asian and white women (*p* < .01, data not shown), which is consistent with two recent studies that have found more robust cortical bone properties in Asian subjects compared with whites.(31,32) Finally, it is possible that the anthropomorphic characteristics of the cohorts in this study (particularly between male and female) are confounding factors for the densitometric, geometric, and biomechanical comparison. Since height and weight data were not available for all subjects, it was not possible to correct for these factors statistically.

Postmenopausal bone loss and response to pharmacologic intervention are manifested by heterogeneous changes in bone geometry (eg, cortical thinning, increased porosity, trabecular thinning, loss of connectivity, etc.). In the context of a clinical osteoporosis study, cortical microstructural properties should be considered complementary to other geometric measures of bone quality such as cross-sectional geometry and trabecular structure. In particular, this collective information could be exploited to inform optimal treatment strategies based on the known action of the pharmacologic agent and to provide an avenue for customized investigations into the structural component of treatment efficacy and fracture risk. It should be noted that the structural and biomechanical techniques developed in this study assume fixed, homogeneous material properties and therefore reflect geometry alone. There is evidence in the literature that sustained treatment with common therapeutic agents is associated with changes in bone matrix composition(33) and possibly changes in the prevalence of microdamage.(34) Changes in bone strength attributable to such material changes cannot be accounted for in the cortical structure and biomechanical analysis described here. Therefore, the ability of these indices to differentiate fracture and their relative significance in characterizing response to therapy require future investigation in appropriate case-control studies.

In conclusion, our results demonstrate that cortical porosity in the distal radius and tibia, as measured by HR-pQCT, exhibits significant age-related differences and differentiate pre- and postmenopausal women. Furthermore, our modeling results suggest that the increase in intracortical porosity is associated with increasingly significant deficits in apparent-level mechanical properties but minimal differences in cortical/trabecular load distribution. Collectively, these findings suggest that HR-pQCT-derived measures of intracortical porosity may be useful quantitative endpoints for bone quality studies involving fracture discrimination and drug efficacy.
